# Chromosome Specific Substitution Lines of *Aegilops geniculata* Alter Parameters of Bread Making Quality of Wheat

**DOI:** 10.1371/journal.pone.0162350

**Published:** 2016-10-18

**Authors:** Monika Garg, Hisashi Tsujimoto, Raj Kumar Gupta, Aman Kumar, Navneet Kaur, Rohit Kumar, Venkatesh Chunduri, Nand Kishor Sharma, Meenakshi Chawla, Saloni Sharma, Jaspreet Kaur Mundey

**Affiliations:** 1 National Agri-Food Biotechnology Institute, Mohali-160071, Punjab, India; 2 United Graduate School of Agriculture, Tottori University, Tottori, Japan; 3 Indian Institute of Wheat and Barley Research, Karnal, India; Institute of Genetics and Developmental Biology Chinese Academy of Sciences, CHINA

## Abstract

Wheat cultivars with wide introgression have strongly impacted global wheat production. *Aegilops geniculata* (M^g^U^g^) is an important wild relative with several useful traits that can be exploited for wheat improvement. Screening of *Ae*. *geniculata* addition lines indicated a negative effect of 1U^g^ and the positive effect of 1M^g^ chromosome on wheat dough strength. Negative effect of 1U^g^ is probably associated with variation in number and position of the tripeptide repeat motif in the high molecular weight glutenin (HMW-G) gene. To utilize the positive potential of 1M^g^ chromosome, three disomic substitution lines (DSLs) 1M^g^(1A), 1M^g^(1B) and 1M^g^(1D) were created. These lines were characterized for morphological, cytogenetic properties and biochemical signatures using FISH, 1D-, 2D-PAGE and RP-HPLC. Contribution of wheat 1A, 1B and 1D chromosomes towards dough mixing and baking parameters, chapatti quality, Fe/Zn content and glume color were identified. Observed order of variation in the dough mixing and baking parameters {1M^g^(1D) ≤wheat ≤1M^g^(1B) ≤1M^g^(1A)} indicated that chromosome specific introgression is desirable for best utilization of wild species’ potential.

## Introduction

Among the cereals, wheat (*Triticum aestivum* L., 2n = 42; AABBDD) is the most widely cultivated crop in the world. Its technological properties are critical for product specific utilization. Globally, a relatively narrow genetic base hampers wheat improvement by limiting its end use quality. Useful genetic variation can be transferred to wheat from its related wild species that act as an excellent reservoir of a number of economically important genes. One such wild relative is tetraploid *Aegilops geniculata* (2n = 28; M^g^M^g^U^g^U^g^) [1,2]. One of the most important traits of *Ae*. *geniculata* is its ecological adaptability. It exhibits high genetic variation [3,4] and synteny with wheat [5] that is useful for its exploitation for improving wheat cultivars. In order to utilize its trait specific potential, different genetic materials, in the form of addition, substitution, and translocation lines had been created [6,7,8].

Considerable research had been conducted on wheat, to uncover the genetic basis of superior bread quality traits such as dough mixing properties, viscoelasticity, loaf volume and loaf score [9,10]. Mixture of wheat proteins called gluten is the major determinant of dough viscoelasticity, forming a network in the dough that confers the viscoelasticity necessary for the production of high-quality bread with a light and porous crumb structure. Gluten consists of two main components- gliadins and glutenins. Gliadins are single chain proteins and are responsible for dough extensibility. Glutenins are polymeric proteins and are classified as high molecular weight glutenin subunits (HMW-GSs) and low molecular weight glutenin subunits (LMW-GSs). They are responsible for dough viscoelasticity [11,12]. HMW-GSs are the major determinants of gluten quality. Differences in the allelic compositions of HMW-GSs have major effect on the baking parameters across a wide array of wheat cultivars [13,14,15]. The genes encoding HMW-GSs are clustered on the long arm of homoeologous group-1 chromosomes (i.e., 1A, 1B, and 1D). Their allelic variation is associated with wheat product quality [16]. HMW-GS genes with higher relative expression, longer repetitive domains and extra cysteine residues impart better bread-making properties [17,18]. Different current end uses of wheat are adjusted to existing variation in HMW-GSs. For highly desirable end product quality, there is a need to broaden the genetic variation in HMW-GS genes through wide introgression; perhaps even new end uses can be found. It is a challenge to study the wild relatives of bread wheat for dough mixing and baking properties because of small seeds that are difficult to thresh and flour recovery is poor for analysis. To overcome this problem, we have used disomic addition lines (DALs) of *Ae*. *geniculata* (bigger seeds) in the genetic background of Chinese Spring (CS) wheat. After selecting 1M^g^ addition line as a positive contributor for bread-making quality, we have produced all three chromosome specific substitution lines. Detailed grain quality analysis of these lines was carried out to understand the genetics and influence of homoeologous group-1 chromosomes and their encoded seed storage proteins (SSPs) on wheat processing and product quality.

## Materials and Methods

### Plant material

Ten DALs of *Ae*. *geniculata* (1M^g^, 2M^g^, 4M^g^, 5M^g^, 7M^g^, 1U^g^, 2U^g^, 4U^g^, 5U^g^, 6U^g^) in the genetic background of CS (Friebe *et al*., 1999) were obtained from the germplasm bank for National Bioresource Project-Wheat (TACBOW 0283–0294), Japan [7]. Plants were grown in three replications in raised lines at Tottori University, Japan from 2005–2006 to 2007–2008.

### Generation of disomic substitution lines (DSL)

#### 1. Generation of DSL1Mg(1D)

DSL1M^g^(1D) appeared spontaneously from the progeny of addition line. Screening and selection procedure included grain quality analysis, electrophoresis of glutenin and gliadin proteins and cytological analysis.

#### 2. Generation of DSL1Mg(1A)

For the generation of chromosome specific substitution line, 1M^g^ addition line was crossed with CS genetic stock-nullisomic for chromosome 1A and tetrasomic for chromosome 1B (N1A-T1B) in the year 2007–2008. Seeds of F_1_ plants were harvested in the year 2008, off season. F_2_ seeds were screened to identify DSL1M^g^(1A). Screening procedure included electrophoresis of glutenin and gliadin proteins and cytological analysis of F_2_ seeds. Selected embryos were transferred to soil to raise F_2_ plants (year 2008–2009). Several F_3_ seeds derived from individual F_2_ plants were screened similarly for the confirmation of DSL1M^g^(1A) F_2_ plants. Confirmed F_3_ seeds were sown and F_4_ seeds were harvested in the subsequent off season.

#### 3. Generation of DSL1Mg(1B)

For generation of DSL1M^g^(1B) substitution line, CS genetic stock-N1B-T1D was crossed with DSL1M^g^(1D) (year 2007–2008). F_2_ seeds were raised from F_1_ seeds in year 2008 off season and screened to identify DSL1M^g^(1B). Further, the F_3_ seeds were raised in the same field in year 2008–2009 and screened for confirmation of the DSL1M^g^(1B) F_2_ plants. F_3_ seeds from the confirmed F_2_ plants were sown to obtain F_4_ seeds in the 2009 off season.

Replicated sowing of F_4_ seeds of all three substitution lines and CS was carried out in the field and greenhouse, at the International Center for Agricultural Research in Dry Areas (ICARDA), Syria in the fifth year (2009–2010). F_5_ seeds thus obtained were verified by rheological analysis at ICARDA. The seeds of CS and substitution lines were regrown in three replications in 2 meter rows (minimum one row) at the farms of National Agri-Food Biotechnology Institute (NABI), India in the sixth year (2010–2011, F_5-6_ seeds), seventh (2011–2012, F_6-7_ seeds), eighth (2012–13, F_7-8_ seeds) and ninth year (2013–14, F_8-9_ seeds). F_7_ seeds were used for bread-making quality analysis at Punjab Agricultural University (PAU), Ludhiana, Punjab, India. F_9_ seeds were used for bread- and biscuit-making quality analysis at Directorate of Wheat Research (DWR), Karnal, Haryana, India and at NABI, Mohali, Punjab, India. All the tests were carried out with three biological and three technical replicates.

### Protein characterization

The seeds of CS, addition and substitution lines were characterised for different parameters. Total seed storage proteins were extracted according to Smith and Payne [19] by using SDS buffer (2% SDS, 10% glycerol, 50 mM DTT, 40 mM Tris–Cl, pH 6.8). Sequential extraction of unreduced gliadins (1.5M dimethyl formamide) followed by reduced glutenins was carried out from the single seeds for screening of substitution lines. Electrophoresis of total proteins and glutenin was carried out using 10% polyacrylamide gel. Gliadins were separated on 17.5% gel [19, 20]. Two-dimensional polyacrylamide gel electrophoresis (2D-PAGE) of total seed proteins was carried out according to Kamal et al. [21]. Briefly sample solutions were loaded onto the acidic side of the isoelectric focusing gel (pH 3–10) for the first dimension. SDS-PAGE in the second dimension was performed with 5% stacking and 10% separating gels. Each sample was run three times, and the best visualized gels were selected. Reversed phase high performance liquid chromatography (RP-HPLC) of separated gliadin and glutenin proteins was carried out according to Mejias et al. [22] with some modifications. The proteins were separated using C8 reversed phase analytical column Zorbax 300SB-C8 (Agilent Technologies, California, USA) with Agilent 1260 infinity quaternary liquid chromatography system. Injection volume was 20 μl and column temperature was set 60^°^C. A linear gradient was set up using solvents A (0.1% TFA in HPLC grade water) and B (0.1% TFA in Acetonitrile). Glutenins were separated at a linear gradient 0% to24% B for 20 minutes and then 24% to 60% B for 40 minutes. Gliadins were separated from 20% to 60% B for 60 minutes. The peaks were detected at 210 nm by diode array UV-Vis detector.

### Cloning and sequencing of HMW-GS genes

Full length HMW-GS genes were amplified [20], cloned, and sequenced from both termini. The NCBI-BLAST was used for sequence comparison. The complete ORFs were determined by overlapping the sequences of the sub-clones created by the nested deletion method [23]. Phylogenetic tree using full length and conserved sequences was constructed by utilizing seeded guide tree, HMM profile-profile techniques and mBed-like clustering guide tree options of Clustal Omega program version 1.2.1. Different features of the translated protein were determined (http://protcalc.sourceforge.net/).

### Grain Quality analysis

During the first year, grain testing of stable addition lines was carried out at Tottori University, Japan. Protein content was measured by near infrared (NIR) spectroscopy. Small-scale sodium dodecyl sulfate-sedimentation (SDSS) values were measured and specific sedimentation was calculated [24,25].

During the second year, grain quality analysis of DAL1M^g^ and CS was performed at the National Agricultural Research Center for Western Region (NARCWR), Fukuyama, Japan. American Association of Cereal Chemists (AACC, 1990) methods [26] were followed for evaluation of moisture, ash and protein content. Size-exclusion high-performance liquid chromatography (SE-HPLC) was carried out for determination of unextractable polymeric proteins [27]. Briefly, for extractable proteins, the flour suspension (10 mg of flour in 1.0 ml of 0.5% w/v SDS-phosphate buffer, pH 6.9) was stirred for 5 min (without sonication) and the supernatant was collected. For unextractable proteins, the pellet was suspended in SDS-phosphate buffer and sonicated for 30 sec at 30% amplitude. SE-HPLC was performed on a Shimadzu LC-10A system (Tokyo, Japan) using TSKgel G4000SW column (Tokyo, Japan). Separation was achieved in 40 min by loading 20 μl of the sample into a 50% (v/v) acetonitrile eluant containing 0.05% (v/v) triflouroacetic acid at a flow rate of 0.5 ml/min. The elution profile was divided into peaks 1 and 2, corresponding to polymeric protein and monomeric protein, respectively. The single kernel characterization system (SKCS) was used to measure grain hardness and related characteristics. The SDSS test was performed for dough strength estimation [24]. Ten gram mixograph was used for determination of dough physical properties [26].

During the fifth year, grain quality analysis of the F_5_ seeds of the substitution lines was carried out at ICARDA, Syria. The AACC (1990) [26] methods were followed for gluten index and farinographic studies. In the seventh year, grain quality analysis of the F_7_ seeds was carried out at PAU, India. Grain characteristics were determined by standard laboratory procedures. Bread baking was carried out according to remixing procedure of Irrivine and McMullan [28]. In the ninth year bread-making, biscuit-making, gluten index and grain characteristics of the F_9_ seeds of same material were studied at DWR, India, following AACC (1990) methods [26]. Chapatti-making was studied according to Mehta et al. [29]. Grain Iron and Zinc levels were estimated by ICP-MS according to AOAC (2000) method [30]. Gliadin, glutenin ratio (gliadin/glutenin) was calculated after sequential extraction of gliadins and glutenins. The ratio of HMW-GSs and LMW-GSs (HMW-GS/LMW-GS) was calculated after electrophoretic separation of glutenins. Statistical testing of studied parameters was carried out with at least three biological and technical replicates.

### Cytogenetic analysis

Genomic *in situ* hybridization (GISH) with genomic DNA of *Ae*. *geniculata* as the probe was performed for confirmation of 1M^g^ chromosome [20]. The identification of wheat 1B chromosomes was carried out by FISH using 45S rDNA (1B- and 6B-specific) as a probe. The identification of wheat 1D chromosome was carried out by GISH with genomic DNA of *Ae*. *tauschii* (D) and FISH with pAs1 (D genome specific sequences) [31]. Total genomic DNA of *T*. *durum* (AB) was used as the blocking DNA.

### Statistical analysis

To minimize the environmental and inter-laboratory variations and to determine the level of significance of the different parameters between the lines, analysis of variance (ANOVA) or analysis of covariance (ANCOVA) was carried out using the Stat view program.

## Results

### Study of ten disomic addition lines

**Fig 1:**
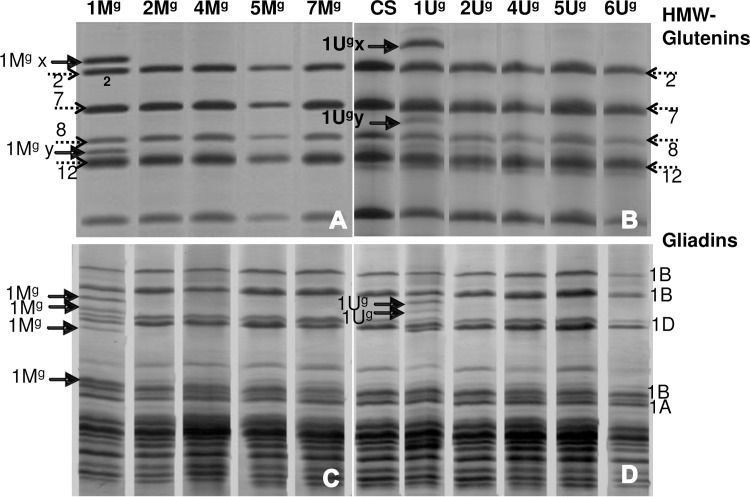
Seed storage protein profiles of different DALs of *Ae*. *geniculata*. A & B; HMW-GS profile, C & D; gliadin profile. DAL1M^g^ and DAL1U^g^ revealed additional HMW-GSs and gliadins (indicated by arrows).

Ten DALs of *Ae*. *geniculata* (1M^g^, 2M^g^, 4M^g^, 5M^g^, 7M^g^, 1U^g^, 2U^g^, 4U^g^, 5U^g^, 6U^g^) were used for the initial screening. Glutenin profiles of ten DALs indicated two additional HMW-GSs each in DAL1M^g^ and DAL1U^g^ (Fig 1). These two selected lines were utilized for further analysis.

In case of DAL1M^g^, additional bands of chromosome 1M^g^ included slow moving band above 1Dx2, and fast moving above 1Dy12 (Fig 1A), indicating longer proteins of *Glu-M*^*g*^*1* than *Glu-D1*. Migration rates of the *Glu-U*^*g*^*1* encoded subunits were even slower than *Glu-M*^*g*^*1* encoded subunits (Fig 1B) indicating longer proteins than *Glu-M*^*g*^*1* and *Glu-D1*. Gliadin protein profiles indicated three additional bands in DAL1M^g^ (Fig 1C) and two additional bands in DAL1U^g^ (Fig 1D) with migration rates closer to 1D encoded ω-gliadins. One additional subunit was observed in DAL1M^g^ closer to chromosome 1B encoded α-gliadins of CS.

### Sequence analysis of HMW-GS genes of *Ae*. *geniculata*

**Fig 2:**
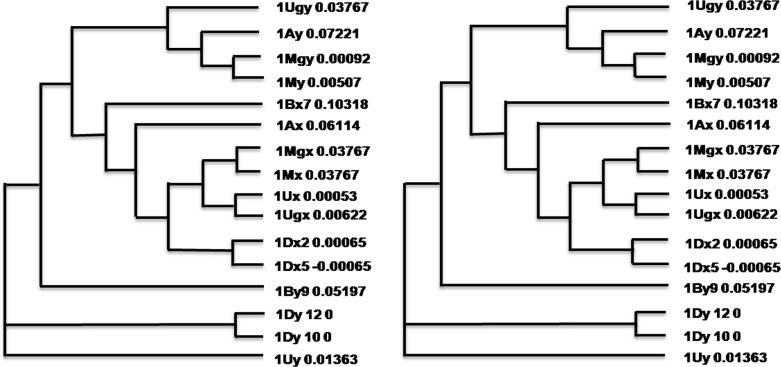
Maximum likelihood tree obtained using neighbor-joining method by Clustal O program 1.2.1. A- Full length sequence, B- Conserved sequences, including signal peptide, N- and C-terminal domain. 1M^g^x aligned with 1Mx, 1U^g^x, 1Ux and 1Dx, 1U^g^x aligned with 1Ux, 1M^g^x, 1Mx and 1Dx, 1M^g^y aligned with 1My and 1Ay and 1U^g^y with 1Ay, 1M^g^y 1My sequences. Database accession numbers of wheat HMW-GSs 1Ax, 1Bx7, 1Dx2, 1Dx5, 1Ay, 1By9, 1Dy12 1Dy10, 1Ux, 1Uy, Mx, My used for comparison are M22208, X03346, X13927, X12928, X03042, X61026, X03041 X12929, AF476961.1, AF476962.1, AY455789.1, and AY455788.1, respectively.

*Ae*. *geniculata* (M^g^M^g^U^g^U^g^) HMW-GS sequences were compared with wheat and related wild species. Sequences showing a better match with HMW-GS genes of *Ae*. *comosa* (MM), were selected to represent 1M^g^x (KX375404) and 1M^g^y (KX375405) sequences. Those showing a better match with *Ae*. *umbellulata* (UU), were selected to represent 1U^g^x (KX375406) and 1U^g^y (KX375407) sequences. Analysis of the derived amino acid sequences indicated typical primary structure as previously reported with conserved signal peptide, N- and C-terminal regions, central repetitive domain and a similar number of cysteine residues (S1 Table). Phylogenetic analysis of full length (Fig 2A) and conserved (Fig 2B) amino acid sequences with wheat, *Ae*.*comosa* and *Ae*. *umbellulata* revealed that 1M^g^x aligned with 1Mx, 1U^g^x, 1Ux and 1Dx, 1U^g^x aligned with 1Ux, 1M^g^x, 1Mx and 1Dx, 1M^g^y aligned with 1My and 1Ay and 1U^g^y with 1Ay, 1M^g^y 1My sequences.

Calculated molecular weight (MW) and isoelectric point (pI) of 1U^g^x, 1U^g^y, 1M^g^x, and 1M^g^y sequences were 103653, 86330, 68374 and 64038 Da and 4.8, 5.9, 6.3 and 6.9, respectively, indicating longer and acidic *Glu-U*^*g*^*1* HMW-GSs. The 1U^g^x and 1U^g^y sequences had a higher proportion of tripeptide repeat motifs, especially EQQ, compared to uniformally distributed nonapeptide and tripeptide repeat motifs in 1M^g^x, 1M^g^y and wheat sequences. 1U^g^y had a higher proportion of glutamic acid/glutamine (0.11) compared to 1M^g^y (0.09) and wheat sequences (0.07). In case of x-type genes, it was uniform (0.056) with an exception of 1Ax (0.087). Multiple tripeptide repeats QQQ observed in y-type wheat sequences, was neither found in 1M^g^y nor 1U^g^y sequences. Three nonapeptides unique to wheat had three gutamine residues, (QQQ), while three nonapeptides unique to 1U^g^y had only single glutamine residue (PQL, LRQ, VLQ). Another important and unique observation was deletion of GQQ tripeptide before the cysteine carrying nonapeptide repeat in the repetitive domain close to the C-terminal domain in 1U^g^y and other U-genome HMW-GS sequences (AF476962.1). Hence, a regular sequence of (PGQ*GQQ*GH**C**PTSPQQ) was converted to (PGQGH**C**PTSPQQ).

### Confirmation of grain quality of DAL1M^g^

During the first year, SDSS/E-SDSS of DAL1M^g^ was higher than background cv. CS (1.36 vs. 0.84) and rest of addition lines indicating higher dough strength. DAL1U^g^ showed lower value than CS (S1 Fig). Therefore, DAL1M^g^ was selected for detailed quality analysis. In the second year, DAL1M^g^ maintained significantly higher SDSS/E-SDSS than CS, both in the field conditions (1.33 vs. 0.9, respectively) and in greenhouse (1.25 vs. 0.85). Assessment of DAL1M^g^ in comparison to CS at NARCWR, Japan, indicated significantly higher SDSS, mixing peak height, and peak band width of DAL1M^g^ in comparison to CS (Table 1). There were insignificant differences in thousand kernel weight, protein content, grain moisture content, grain hardness index, particle size index, and starch pasting properties between DAL1M^g^ and CS.

**Table 1:** Rheological parameters of DAL-1M^g^ in comparison to CS (year 2005–2006) at Tottori University, Japan.

Line	TKW	Protein Content (%)	Moisture Content (%)	Grain Hardness Index	Sedimentation Value (ml)	Mixograph		
						MPW	MPT	MPH
**DAL1M**^**g**^	33.3±2.3	13.7±1.6	11.2±0.3	44.6±3.6	32.0±1.0^b^	22.0±1.0^b^	1.2±0.1	48±0.6^b^
**C/S**	32.0±1.4	12.3±2.1	11.1±0.5	41.3±6.2	23.4±1.9^a^	17.3±2.1^a^	1.3±0.1	43.6±0.4^a^

Values followed by the same letter in the same column are not significantly different at ^*p*^<0.05.

MPW-Midline Peak Width, MPT- Midline peak time, MPH- Midline peak height

### Instability of addition line and creation of DSL1M^g^(1D)

**Fig 3:**
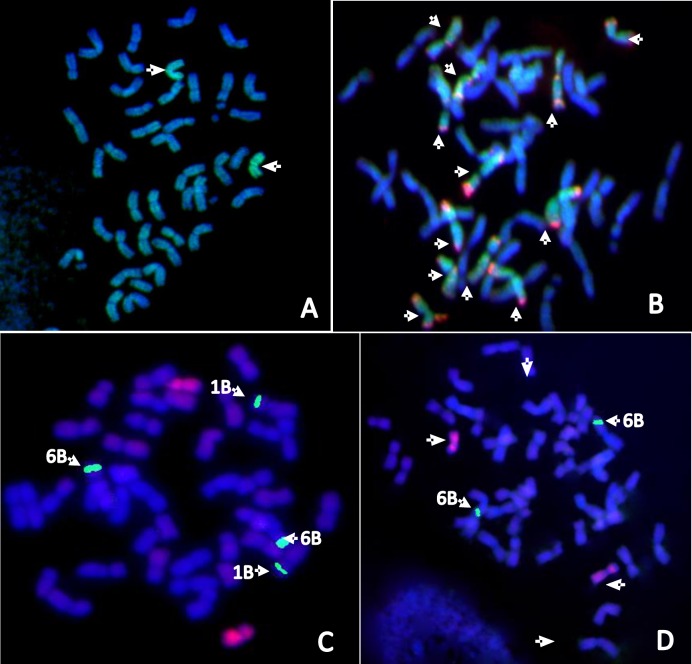
GISH/FISH images of mitotic chromosomes. A- DSL1M^g^(1D), B- DSL1M^g^(1D), C- DSL1M^g^(1A) and D- DSL1M^g^(1B). GISH revealed two *Ae*. *geniculata* chromosomes and 12 instead of 14 D-genome chromosomes in DSL1M^g^(1D). The DSL1M^g^(1A) was identified from total 42 chromosomes, two *Ae*. *geniculata* chromosomes and four 45srDNA signals. The DSL1M^g^(1B) was identified from total 42 chromosomes, two *Ae*. *geniculata* chromosomes and two 45SrDNA signals.

During the third year, several DAL1M^g^ replicates from the field as well as greenhouse had lower SDSS/E-SDSS values than CS. The SDS-PAGE profile indicated the presence of *Glu-M*^*g*^*1* but absence of *Glu-D1* encoded HMW-GSs in three of the four replications from the field and two of the four replications from the greenhouse (S2 Fig, arrowheads). All these replicates had lower SDSS/E-SDSS values than CS and were taken as representative 1M^g^(1D) disomic substitution lines DSL1M^g^(1D). Another replicate, with normal expression of *Glu-D1* encoded subunits, had significantly higher SDSS/E-SDSS values than CS (S2 Fig). It was taken as representative DAL. GISH of the mitotic chromosome preparations from the root tip cells of representative DSL1M^g^(1D) revealed two *Ae*. *geniculata* chromosomes (Fig 3A). Further GISH/FISH analysis using genomic DNA of *Ae*. *tauschii* (D-genome donor) and pAs1 (D-genome specific sequences) revealed twelve D-genome chromosomes rather than regular fourteen in DSL1M^g^(1D) (Fig 3B). pAs1 signals identified missing chromosome pair as 1D. Therefore representative substitution lines were confirmed as DSL1M^g^(1D).

Grain and flour properties of DSL1M^g^(1D) were similar to CS. Mixograph properties showed significant decrease in mixing peak time in comparison to CS (Table 2). SE-HPLC analysis of greenhouse samples indicated significantly higher and lower polymeric protein content (UPP/TPP%) in the addition and substitution lines, respectively, in comparison to CS.

**Table 2:** Rheological parameters of DSL1M^g^(1D) in comparison to CS (year 2006–2007) at Tottori University, Japan.

Line	Protein Content (%)	Sedimentation value (ml)	Mixograph			UPP/TPP (%)
			MPW	MPT	MPH	
**1Mg**	-	-	-	-	-	51.7±1.6^c^
**1Mg(1D)**	12.3±0.3	23.7±1.2	16.1±2.0	0.5±0.2^a^	43.2±0.7	36.8±1.3^a^
**CS**	12.6±0.7	24.2±0.8	16.3±1.1	1.4±0.1^b^	43.0±0.4	40.8±1.8^b^

Values followed by the same letter in the same column are not significantly different at ^*p*^ <0.05.

MPW-Midline Peak Width, MPT- Midline peak time, MPH- Midline peak height, UPP- Unextractable polymeric protein, TPP- Total polymeric protein. The missing values are due to unavailability of addition line seeds where elimination of chromosome 1D resulted in subsequent conversion of addition line to substitution line.

### Generation of substitution lines

To utilize the potential of chromosome 1M^g^ coded HMW-GSs for improvement of wheat bread making quality, substitution lines of chromosome 1A {DSL1M^g^(1A)} and 1B {DSL1M^g^(1A)} were created.

**Fig 4:**
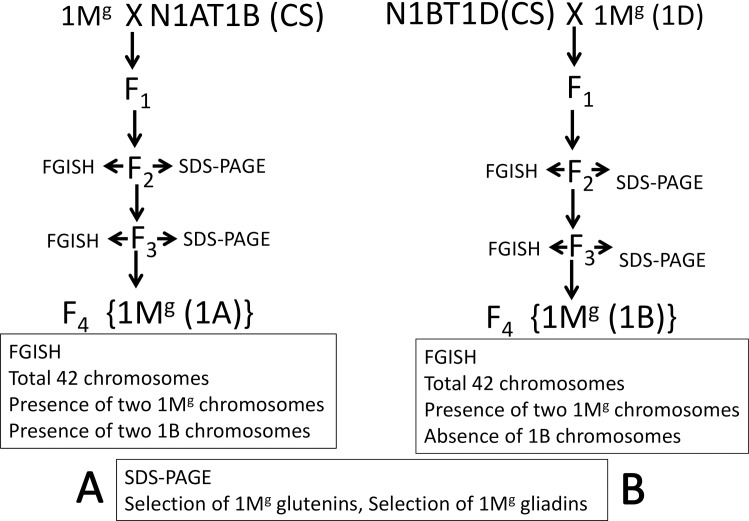
Development of different substitution lines. A- DSL1M^g^(1A), B- DSL1M^g^(1B). DSLs were prepared by crossing an addition/substitution line with nulli-tetra genetic stock followed by selection using storage protein profile and cytological analysis.

To generate DSL1M^g^(1A), DAL1M^g^ was crossed with N1A-T1B (Fig 4A). The F_1_ plants were double monosomic for chromosome 1M^g^ and 1A and trisomic for 1B. Endosperm half of the F_2_ seeds were screened for the presence of 1M^g^ chromosomes by HMW-GS profile and absence of 1A by gliadin profile. Of the 20 seeds analyzed, 14 carried the 1M^g^-encoded HMW-GSs. Out of these 14, two selected F_2_ seeds revealed the absence of chromosome 1A-encoded gliadins. Selected embryos were grown in petri plates and 1–2 roots out of 3 were fixed for cytological analysis. GISH revealed single *Ae*. *geniculata* chromosome in first and two in second embryo. FISH with the 45S rDNA probe (specific for 1B and 6B chromosome pairs) gave six signals (6B-2, 1B-4) in first and four (6B-2, 1B-2) in second plant. The second plant with total 42 chromosomes, two each of 1M^g^ and 1B and missing 1A was selected and grown to raise the F_3_ seeds (Fig 3C). Uniform SDS-PAGE and FISH profiles of all the analyzed F_3_ seeds confirmed F_2_ plant as a stable substitution line {DSL1M^g^(1A)}.

To generate DSL1M^g^(1B), CS genetic stock N1BT1D was crossed with DSL1M^g^(1D) (Fig 4B). Later was preferred over DAL1M^g^ with the idea that extra 1D chromosome in N1BT1D gametes would be balanced by the absence of 1D chromosome in DSL1M^g^(1D) gametes. F_1_ plants would only be double monosomic for chromosomes 1M^g^ and 1B. Screening for DSL1M^g^(1B) was initiated at F_2_ stage. Out of the 20 analyzed seeds, 7 carried the 1M^g^-encoded HMW-GSs as well as gliadins and did not carry chromosome 1B encoded glutenins and gliadins. GISH analysis using *Ae*. *geniculata* genomic DNA as probe revealed single *Ae*. *geniculata* chromosome in four and two in three embryos. Fluorescence *in situ* hybridization (FISH) analysis with the 45S rDNA probe (Fig 3D) gave two signals only (6B) indicating absence of 1B chromosomes in all the selected seeds. Three plants which contained, total 42 chromosomes with the presence of two 1M^g^ instead of 1B chromosomes (Fig 3D) were selected and grown to raise the F_3_ seeds. Uniform SDS-PAGE and the FISH patterns of all the analyzed F_3_ seeds from two F_2_ seeds, confirmed the stable substitution line. F_3_ seeds of selected F_2_ plants were sown to multiply seeds for further analysis.

### Analysis of the *Ae*. *geniculata* substitution lines

Substitution lines were characterized by analyzing their morphological, biochemical, rheological and baking properties and by understanding the influence of *Ae*. *geniculata* as well as wheat homoeologous group-1 chromosomes on wheat technological properties.

### Morphological traits of DSLs

**Fig 5:**
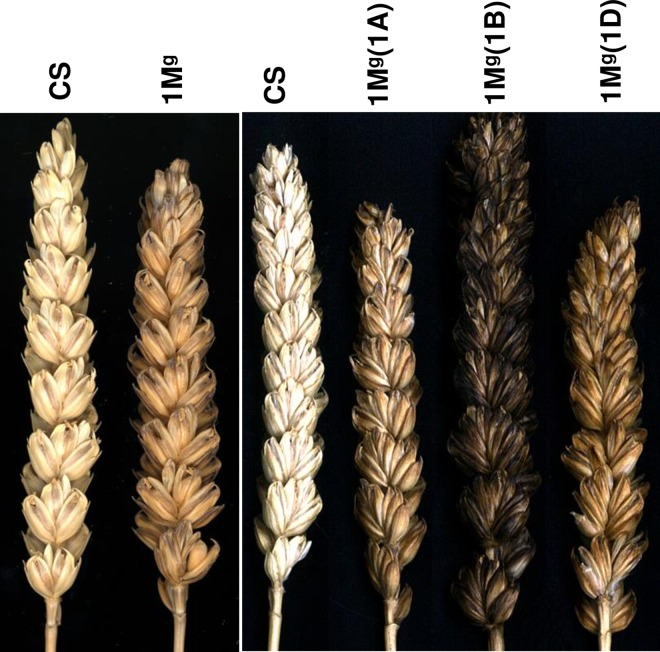
Spike morphology of CS, DAL1M^g^ and DSLs. Glume color of DSL1M^g^(1B) was darker than other tested lines.

Addition as well as substitution lines of *Ae*. *geniculata* were morphologically similar to CS. DSL1M^g^(1D) flowered earlier than others. DAL1M^g^ and all DSLs had black glume whereas, DSL1M^g^(1B) had darkest glume among the tested lines (Fig 5).

### Seed storage protein profile (1D-PAGE) of DSLs

**Fig 6:**
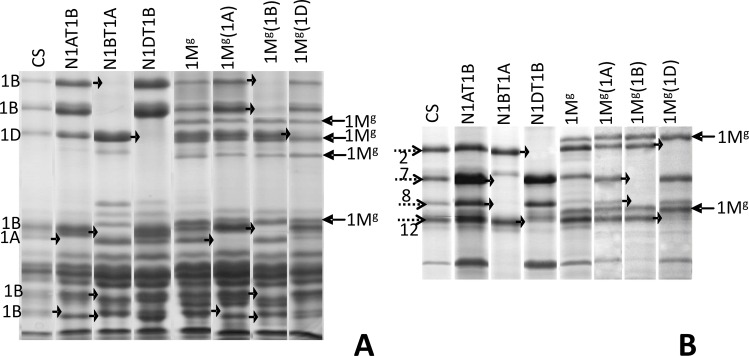
Seed storage protein profile of CS, DAL1M^g^ and DSLs. A- Gliadins, B-HMW-GSs. Nulli-tetra lines were used for comparison. New subunits are indicated by arrows and deleted subunits by arrowheads.

DAL1M^g^ and DSL1M^g^(1A) had six HMW-GSs, four from CS (1Dx2, 1Dy12, 1Bx7, 1By8) and two from *Ae*. *geniculata* (1M^g^x, 1M^g^y, Fig 6B). DSL1M^g^(1B) {1Dx2, 1Dy12, 1M^g^x, 1M^g^y} as well as DSL1M^g^(1D) {1Bx7, 1By8, 1M^g^x, 1M^g^y} had four HMW-GSs. Gliadin profile (Fig 6A) indicated additional subunits of *Gli-M*^*g*^*1* gliadin locus in the addition and substitution lines (3 in the ω-gliadin and 1 in α-gliadin region). DSL1M^g^(1A) showed two missing bands in the α-gliadin range. DSL1M^g^(1B) had three missing bands in α-gliadin and two in ω-gliadin region. DSL1M^g^(1D) had one missing band each in the α-gliadin and ω-gliadin region.

### Seed storage protein profile (2D-PAGE) of DSLs

**Fig 7:**
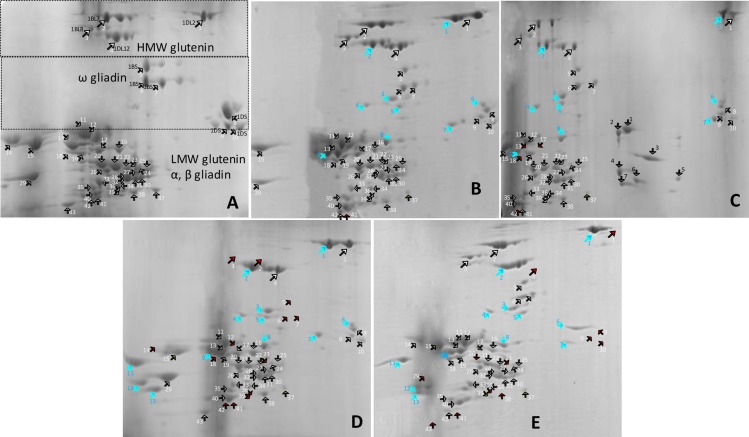
2D-PAGE profile of total proteins. A- CS, B- DAL1M^g^, C- DSL1M^g^(1A), D- DSL1M^g^(1B), E- DSL1M^g^(1D). As indicated, several differences were observed in total protein 2D-PAGE profiles of CS, DAL and DSLs.

Several differences were observed in 2D-PAGE total protein profiles of CS, DAL and DSLs (Fig 7, S3 Table). In CS four HMW-GSs were found (Fig 7A), but 1Dx2 had two and 1Dy12, 1Bx7, 1By9 had three spots with similar MW but different isoelectric point (pI) that may indicate different modified forms e.g., glycosylated or phosphorylated. In the DAL1M^g^, 1M^g^x with two spots had an acidic pI near 1Dx2 and 1M^g^y with three spots had a higher pI near 1Bx7 (Fig 7B). In the DSL1M^g^(1B) and DSL1M^g^(1D) corresponding wheat HMW-GSs were missing (Fig 7D and 7E). In DSL1M^g^(1A) all the HMW-GSs were identified (Fig 7C). In CS, ω-gliadin range, three *Gli-B1* gliadin spots had higher pI than three *Gli-D1* spots. In DAL and DSLs, five additional *Gli-M*^*g*^*1* ω-gliadin spots, three in higher and two in lower pI range were observed. In LMW-GS, α-, γ-gliadin range, CS showed 33 spots with five additional spots of 1M^g^ in DAL and DSLs. In the same region, DAL1M^g^, DSL1M^g^(1A), DSL1M^g^(1B) and DSL1M^g^(1D) revealed 1, 4, 8 and 7 missing and 1, 1, 2, 4 relatively reduced expression bands, respectively.

### Seed storage protein profile-RP-HPLC of DSLs

**Fig 8:**
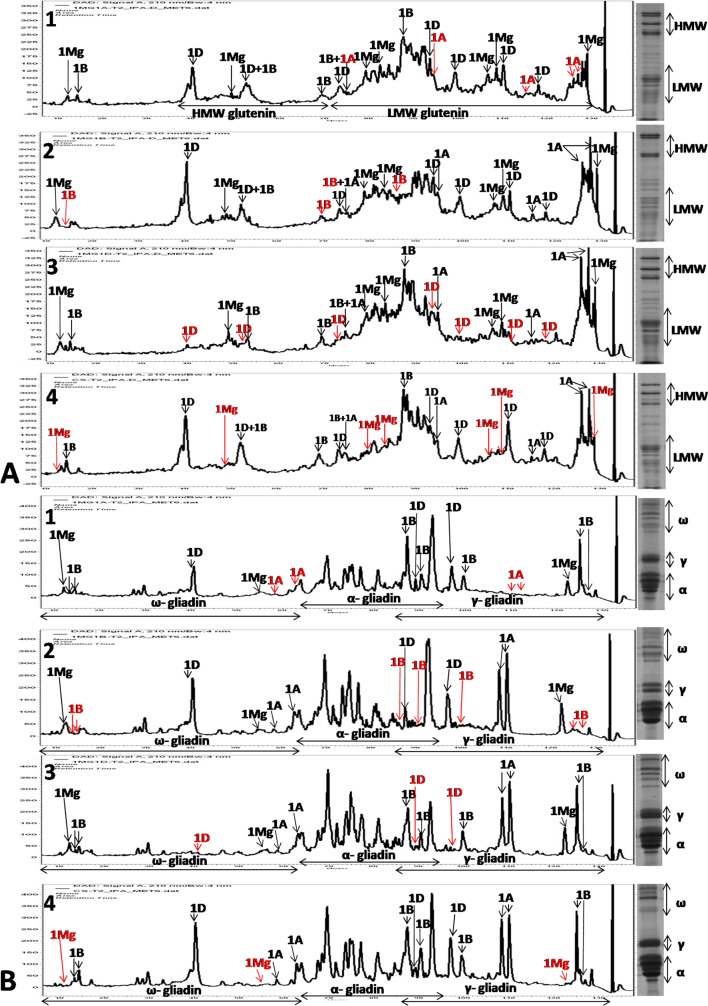
RP-HPLC profiles of DSLs in comparison to CS. A- glutenins, B- gliadins. 1- DSL1M^g^(1A), 2- DSL1M^g^(1B), 3- DSL1M^g^(1D), 4-CS. Almost all the peaks were assigned to individual wheat or *Ae*. *geniculata* chromosomes.

RP-HPLC of glutenins and gliadin proteins of CS and DSLs was carried out to estimate their relative expression. Glutenin profile indicated two 1D coded, single 1B coded and single 1M^g^ coded HMW-GS peaks. Second 1B and 1M^g^ coded peaks observed in 1D- and 2D-PAGE could not be separated in the RP-HPLC (Fig 8A). LMW glutenin region was better separated in the RP-HPLC profile. Almost all peaks were assigned to individual 1A, 1B, 1D and 1M^g^ chromosomes. Out of the total 18 peaks identified in the LMW glutenin region, 5 peaks were assigned to chromosome 1A, 3 to chromosome 1B, 5 to chromosome 1D and 5 to chromosome 1M^g^. RP-HPLC of gliadins indicated two peaks each coded by 1M^g^, 1A, 1B and 1 peak coded by 1D chromosome in the ω-gliadin region (Fig 8B). The α-, γ- gliadin region was better separated in the RP-HPLC profile. Almost all peaks of γ- gliadins were assigned to individual 1A, 1B, 1D and 1M^g^ chromosomes (Fig 8B). Out of the total 10 peaks identified in the γ- gliadin region 2 peaks were assigned to chromosome 1A, 5 to chromosome 1B, 2 to chromosome 1D and 1 to chromosome 1M^g^. LMW-GS and α-, γ- gliadin region could be better separated by RP-HPLC while HMW-GS and ω-gliadin region by SDS-PAGE. Lower expression of 1D coded ω and all α-gliadins was observed in DSL1M^g^(1A) that might be the reason for the lower gliadin/glutenin ratio in DSL1M^g^(1A) as compared to CS and other DSLs.

### Grain quality parameters of DSLs

Farinographic studies on field grown (ICARDA, Syria) seeds indicated significantly reduced dough strength in DSL1M^g^(1D), than CS as revealed by lower dough development time (DDT), stability time (ST) and higher softening (Table 3). DSL1M^g^(1A) had highest dough strength, as revealed by higher DTT, ST and lower softening. Dough strength of DSL1M^g^(1B) was higher than CS but lower than DSL1M^g^(1A). Protein content of DSLs was similar to CS. Their seeds were smaller and harder than CS. Similar results were obtained from the greenhouse grown samples (S2 Table).

**Table 3:** Rheological parameters of field grown DSLs in comparison to CS (2009–2010) at ICARDA, Syria.

Line	TKW	Protein	PSI	FAB	FDT	FST	softening
	g	%	%	%	min	min	FU
**1Mg (1A)**	19.5±1.2^a^	15.8±0.4	55.0±0.5^b^	59.5±0.3	4.0±0.1^d^	6.5±0.6^d^	46.0±2.6^a^
**1Mg (1B)**	19.8±1.2^a^	16.5±0.6	59.0±0.2^c^	60.5±1.2	2.7±0.3^c^	3.5±0.3^c^	43.0±3.1^a^
**1Mg (1D)**	19.9±0.6^a^	15.8±1.1	54.0±0.4^b^	58.0±0.9	1.0±0.5^a^	1.2±0.2^a^	105.0±7.2^c^
**CS**	21.7±0.7^b^	16.0±0.8	40.3±1.1^a^	59.0±0.8	1.7±0.2^b^	2.6±0.4^b^	53.0±2.2^b^

Values followed by the same letter in the same column are not significantly different at ^*p*^ <0.05.

PSI-Particle size index, FAB- Farinograph absorbance, FDT- Farinograph development time, FST- Farinograph stability time, FU- Farinograph units

In the subsequent tested year, (Punjab, India) gluten index of DSL1M^g^(1B) and DSL1M^g^(1D) was lower than CS (Table 4). Gluten index of DSL1M^g^(1A) was higher than CS and other DSLs. Bread loaf studies indicated significantly higher loaf volume as well as loaf score in DSL1M^g^(1A) compared to CS and rest of substitution lines. Differences were observed in the environmentally controlled traits such as protein content, hardness and test weight. The protein content and hardness of DSL1M^g^(1D) and CS were lower than DSL1M^g^(1A), DSL1M^g^(1B). The test weight of former was higher than the later.

**Table 4:** Rheological and baking parameters of DSLs in comparison to CS (2011–2012) at PAU, Ludhiana, India.

**Line**	Grain Appearance	Test Weight	Protein Content	Grain Hardness	Wet Gluten	Dry Gluten	Gluten index	Bread Loaf Volume	Loaf Score	Phenol test
	(Max 10)	(kg/hl)	(%)	(kg)	(%)	(%)		(ml)	(Max 10)	(Max 10)
**1Mg(1A)**	2.8±0.7^a^	66.5±3.2^a^	12.7±0.7^b^	10.4±0.0^c^	29.8±0.^9b^	9.7±0.2^b^	54.6±0.8^c^	505±7.6^b^	5.8±0.1^b^	3.3±0.2
**1Mg(1B)**	2.0±1.1^a^	64.0±2.2^a^	12.2±0.4^b^	10±0.2^b^	32.1±0.5^c^	10.6±0.2^c^	4.4±0.4^a^	460±8.2^a^	5.0±0.3^a^	3.4±0.1
**1Mg(1D)**	4.8±1.2^b^	73.5±3.1^b^	11.6±0.4^a^	9.7±0.3^b^	29.0±0.8^b^	9.8±0.1^b^	4.2±0.5^a^	475±10.2^a^	5.1±0.2^a^	3.2±0.0
**CS**	4.2±0.7^b^	70.5±2.8^b^	11.1±0.5^a^	9.2±0.1^a^	23.3±1.3^a^	9.3±0.1^a^	7.7±0.2^b^	450±9.6^a^	4.5±0.5^a^	3.3±0.1

Values followed by the same letter in the same column are not significantly different at ^*p*^ <0.05.

Third year analysis of field grown lines in Punjab, India (Table 5) revealed insignificant differences in gluten index between DSL1M^g^(1B), DSL1M^g^(1D) and CS. Its value was significantly higher in DSL1M^g^(1A). Bread loaf studies indicated slightly, but significantly higher bread loaf in the DSL1M^g^(1A) compared with the rest of the lines. The loaf score of the line was significantly higher than the rest of the lines. Biscuit making studies indicated higher biscuit spread factor in DSL1M^g^(1A) and DSL1M^g^(1B). The sedimentation value of DSL1M^g^(1D) was lower than CS and other lines. It was highest in DSL1M^g^(1A) and followed by DSL1M^g^(1B). Both these lines had higher sedimentation value than CS. Gliadin/glutenin ration was higher in DSL1M^g^(1D) and lower in DSL1M^g^(1A) in comparison to CS and DSL1M^g^(1B). HMW-GS/LMW-GS was higher in DSL1M^g^(1A) and DSL1M^g^(1B). All lines had similar test weights. Grain hardness and protein content of DSL1M^g^(1A) and DSL1M^g^(1B) were slightly, but significantly lower than CS and DSL1M^g^(1D). Chapatti making studies on the characterized lines indicated higher chapatti score in DSL1M^g^(1D). In particular, this line had better rolling and puffing quality than others. Order of iron content was DSL1M^g^(1A) > DSL1M^g^(1B) >CS = DSL1M^g^(1D) and zinc content was DSL1M^g^(1B) > DSL1M^g^(1A) >CS = DSL1M^g^(1D).

**Table 5:** Rheological and baking parameters of DSLs in comparison to CS (2013–2014) at DWR, Karnal, India.

Cultivar	Test Weight (kg/hl)	Protein Content (%)	Grain Hardness Index	Sedimentation Value (ml)	Wet Gluten (%)	Dry Gluten (%)	Gluten Index	Bread Loaf Volume (ml)	Loaf Score	Biscuit Spread Factor	Chapati score	Gliadin/ Glutenin ratio	HMWGS/ LMWGS	Iron/Zinc
**1Mg(1A)**	74.2±0.5	11.4±0.07^a^	35.0±10^a^	45.5±1.5^d^	25.6±0.9^a^	9.8±0.6^a^	69.5±1.5^b^	550±10^b^	6.2±1.4^b^	10.1±0.7^b^	6.8±0.5^a^	1.9±0.1^a^	1.3±0.02^b^	60.4±5.7^c^ /41.2±2.9^b^
**1Mg(1B)**	72.3±2.2	11.9±0.06^a^	29.5±2.5^a^	42±0.0^c^	27.8±0.7^b^	9.1±0.3^a^	49.5±6.5^a^	535±8^a^	5.5±3.8^a^	10.1±0.2^b^	6.8±0.2^a^	2.6±0.3^b^	1.3±0.1^b^	52.4±6.8^b^ /46.2±3.9 ^c^
**1Mg(1D)**	74.1±0.5	12.9±0.2^b^	41.5±1.5^b^	36.5±3.5^a^	28.3±0.2^b^	10.1±0.7^b^	42.0±5.0^a^	540±10^a^	5.1±1.1^a^	9.2±0.2^a^	7.3±0.3^b^	2.8±0.5^bc^	1.08±0.2^a^	49.7±3.6^a^ /38.2±2.2^a^
**C/S**	71.3±3.1	12.8±0.5^b^	43.1±4.3^b^	39.7±1.8^b^	29.4±1.5^b^	10.7±0.4^b^	47.7±8.8^a^	543±11^a^	5.2±2.4^a^	9.2±0.6^a^	6.7±0.9^a^	2.40±0.4^b^	0.74±0.3^a^	46.9±3.2 ^a^ /36.4±2.6^a^

Values followed by the same letter in the same column are not significantly different at ^*p*^ <0.05.

## Discussion

This study reports positive influence of 1M^g^ chromosome of *Ae*. *geniculata* and negative influence of its 1U^g^ chromosomes on wheat flour mixing properties. Reduction in dough mixing property by 1U^g^ HMW-GSs is expected to be associated with tripeptide repeat motif numbers, sequence, distribution and position in the protein on the basis of sequence comparison. Chromosome specific DSLs were created in this work. Study of DSLs indicated the importance of chromosomes 1A and 1D for dough mixing properties. Baking studies indicated that the potential of 1M^g^ proteins to improve bread making quality can be best utilized by substitution with corresponding chromosome 1A loci.

The contribution of homoeologous group-1 and their HMW-G genes towards bread making quality has been well documented [13,14,32]. After screening 177 DALs belonging to different wild species, we identified *Ae*. *geniculata* DAL1U^g^ and DAL1M^g^ with reduced and improved dough strength. Thus *Ae*. *geniculata* DALs were selected for further study. From ten available *Ae*. *geniculata* addition lines in wheat, we identified DAL1U^g^ and DAL1M^g^ with additional HMW-GSs and gliadins. The HMW-GSs are major determinants of wheat processing quality. In the HMW-GSs number of cysteine residues involved in the intermolecular disulphide bridges and the length of the repetitive domain involved in β-sheet structure and H-bonds have been reported to affect gluten macropolymer and flour technological parameters [33,34,35]. However, both of these could not explain the reduced dough strength in DAL1U^g^ compared to CS, as it carried two additional HMW-GSs with long repetitive domain and typical number of cysteine residues. In this case, it could be better explained with pI, glutamine content, distribution of repeat motif, especially near the cysteine residues involved in intermolecular disulphide bonding [36,37]. The DAL1U^g^_,_ HMW-GS genes had lower pI than corresponding DAL1M^g^ and wheat sequences. More acidic proteins are expected to form a lower number of H-bonds [36]. Higher proportion and poor distribution of tripeptides in repeat motifs in x-type subunit, absence of triple glutamine tripeptides (QQQ) and reduced glutamine content in y-type subunits might have influenced dough strength of DAL1U^g^. Another important observation was absence of GQQ tripeptide before the nonapeptide repeat with cysteine amino acid, in the repetitive domain close to the C-terminal domain. It might be affecting its tertiary structure and therefore availability of cysteine residue for intermolecular bonding with other HMW- and LMW-GSs [38]. The importance of this tripeptide was supported by our similar observations on *Ae*. *umbellulata* chromosome 1U addition line with reduced dough strength, in spite of addition of HMW-GSs with longer repetitive domain and typical number and position of cysteine residues.

Differences in mixing strength and baking performance among different wheat genotypes largely reside in the insoluble glutenin fraction of endosperm proteins [39,40]. Peak mixing time decreases, peak height and peak band width increase with an increase in protein content. All three characters increase with an increase in protein quality [40,41,42]. Selected DAL1M^g^ had improved dough strength than CS as indicated by higher SDSS value, UPP, mixing peak height and mixing peak width with non-significantly different protein content that was maintained across environments. It must be associated with chromosome 1M^g^ coded proteins of *Ae*. *geniculata*. As, among the wheat proteins, polymeric glutenins especially HMW-GSs are major contributors towards dough mixing and baking properties [41,43], therefore it is expected that similar proteins from chromosome 1M^g^ might be contributing towards improved dough strength of DAL1M^g^. The next step of confirmation and utilization of contributing proteins of wild chromosome is to create chromosome specific substitution lines and study their mixing and baking properties. Studies on created DSLs across environments indicated drastic improvement and reduction in mixing parameters in DSL1M^g^(1A) and DSL1M^g^(1D), respectively, with small but statistically significant improvement in DSL1M^g^(1B). It was evident from mixographic and UPP studies in Tottori, Japan, farinographic studies in Aleppo, Syria and gliadin/glutenin ratio, HMW-GS/LMW-GS ratio and gluten index in Punjab and Haryana, India. DSL1M^g^(1D) showed significantly reduced peak mixing time, glutenin macropolymer, farinograph development time, stability time, gluten index and glutenin/gliadin ratio compared to CS. DSL1M^g^(1B) had improved farinograph development time, stability time and HMW-G/LMW-G ratio but with non-significantly different gluten index and glutenin/gliadin ratio. DSL1M^g^(1A) showed drastically improved farinograph development time, stability time, gluten index, glutenin/gliadin ratio and HMW-GS/LMW-GS ratio. Baking studies indicated improved loaf volume and loaf score in DSL1M^g^(1A) with non-significant differences in DSL1M^g^(1B) and DSL1M^g^(1D). Our study highlights the importance of chromosome 1D for wheat mixing and baking parameters. It will be possible to improve technological properties of wheat by wide introgression if transferred to chromosome 1B or 1A. The effect will be a drastic improvement with chromosome 1A specific transfer and significant improvement in 1B transfer [37]. Improved chapatti score with better rolling ability and puffing in DSL1M^g^(1D) is an important observation that can help in understanding different genes involved in relatively less studied chapatti making quality. DSL1M^g^(1D) showed very low gluten index that may be associated with its better chapatti making quality. Improvement in observed biscuit spread factor in DSL1M^g^(1A) and DSL1M^g^(1B) was due to reduced protein content in these lines in the studied year.

Grain size, protein content and hardness show genotype by environment effect [44]. Protein content was non-significantly different between studied lines in Japan and Syria, higher and lower, respectively in first and second studied years in India in DSL1M^g^(1A) and DSL1M^g^(1B) than others, indicating the influence of environment on protein content. Similar environmental influence was observed for grain size and hardness.

Higher genotype effect than the environment effect had been reported in mixing properties than baking qualities. These parameters are usually controlled by several genes with different effects called quantitative trait loci (QTL). The QTLs for the mixographic peak time had been reported on 1DL, 1B, 2D, 2B, 2AL, 7AS and 7DS chromosomes [45,46]. Our observations of DSLs suggested a positive influence of chromosome 1D of CS on mixographic peak mixing time, UPP, farinographic development and stability time and negative influence of chromosome 1A of CS on farinographic development and stability time, gluten index, loaf volume, loaf score and Fe/Zn content.

The genes/QTLs may influence the trait positively, negatively or through epistatic interactions [47,48]. Glume color has been reported to be under the control of *Rg* genes (red glume) located on 1AS, 1BS and 1DS in hexaploid wheat and *Bg* genes (black glume) on 1AS and 1DS in durum and synthetic wheat [49]. Spikes of DAL1M^g^, DSL1M^g^(1D) and DSL1M^g^(1A) has a black glume color, the darkest being in the DSL1M^g^(1B). This was due to black glume color genes associated with chromosome 1M^g^ of *Ae*. *geniculata* [7] that may be a different allele of *Rg* and *Bg* loci in wheat. The primary effect of *Bg-M*^*g*^*1* is conditioned upon the presence of *Bg-B1* locus that probably reduces the effect of *Bg-M*^*g*^*1* indicating conditional epistasis. To the best of our knowledge, due to genome complexity in wheat, a gene specific epistasis effect is being reported here for the first time.

Creation of DSLs is tedious and time consuming. While DSL1M^g^(1D) appeared spontaneously, combination of biochemical protein markers with cytological markers helped in the development of DSLs in three generations only. Combination of different protein separation techniques viz. 1D-PAGE, 2D-PAGE and RP-HPLC helped in assignment and calculation of number of proteins coded by 1A, 1B and 1D chromosomes. The overall number of 1B coded storage proteins was higher than others, with higher number of γ-gliadins and lower number of LMW-GSs. While PAGE detected a lower number of 1A coded proteins, HPLC indicated their expression to be equivalent to 1D and 1M^g^. Improvement in mixing properties in DSL1M^g^(1B) is probably due to reduction in gliadins leading to higher glutenin/gliadin ratio. In the DSL1M^g^(1A) it might be due to increase in number of HMW-GSs i.e., six compared to four in DSL1M^g^(1B) and DSL1M^g^(1D) and creation of better gluten macro-polymer by formation of higher intermolecular disulphide- and H-bonds.

1D-PAGE could effectively separate HMW-GS and ω-gliadins. 2D-PAGE could better separate different proteins compared to 1D-PAGE, but it was more tedious. RP-HPLC was a better technique for the separation of LMW-GSs and α-, γ- gliadins. Since in the present investigation DAL and DSLs were in the soft wheat CS background and is not suitable for bread making quality, therefore, it will be interesting to generate these DSLs in the hard wheat background and study their influence on mixing and baking qualities in wheat.

## Conclusions

DAL1M^g^ of *Ae*. *geniculata* showed positive influence and DAL1U^g^ negative influence on wheat dough strength. Based on sequence comparison, the dough strength reduction by 1U^g^ HMW-GSs is probably associated with the tripeptide repeat motif number, sequence, distribution and position in the gene. Chromosome specific DSLs were created and analyzed. Study of DSLs indicated the importance of chromosomes 1A and 1D for dough mixing properties. The potential of *Glu-M*^*g*^*1* proteins for bread quality improvement can be best utilized by replacement with corresponding chromosome 1A loci of wheat.

## Supporting Information

S1 Fig Comparison of dough strength of different addition lines. (TIF)

S2 Fig Spontaneous generation of DSL1M^g^(1D). Note the missing 1D HMW-GSs and corresponding reduction in specific sedimentation.
(TIF)

S1 Table Comparison of ORF HMW-GS sequences of *Ae*. *geniculata* with wheat and related sequences. (DOCX)

S2 Table Rheological parameters of Green house raised DSLs in comparison to CS (2009–2010) at ICARDA, Syria. (DOCX)

S3 Table List of 2D-PAGE spots indicating present (P), absent (A), reduced expression (R) spots in CS, DAL and DSLs. (DOCX)
