# Supplementary figures and images for: Chromosome Specific Substitution Lines of Aegilops geniculata Alter Parameters of Bread Making Quality of Wheat
Source: PLoS One. 2016 Oct 18;11(10):e0162350. doi: 10.1371/journal.pone.0162350 (PMC5068752; doi:10.1371/journal.pone.0162350)

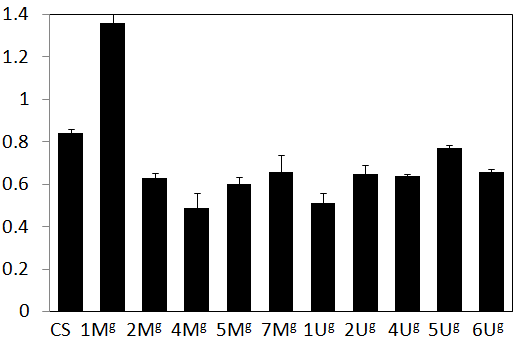

Supplement: S1 Fig — (TIF) [file pone.0162350.s001.tif]

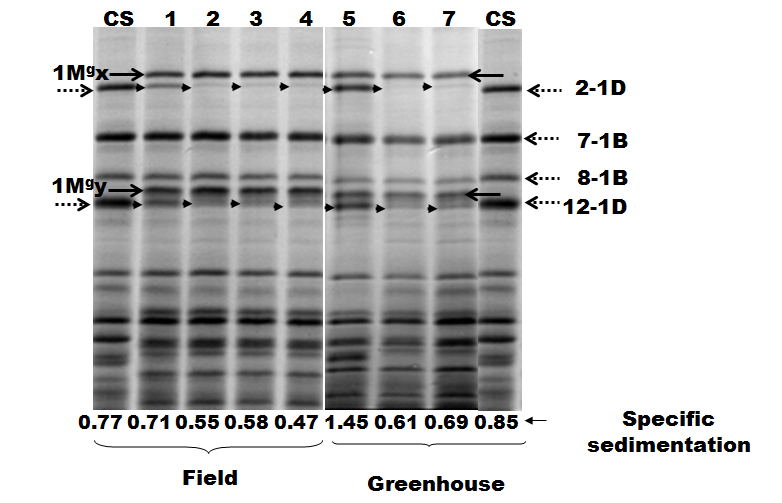

Supplement: S2 Fig — Note the missing 1D HMW-GSs and corresponding reduction in specific sedimentation. (TIF) [file pone.0162350.s002.tif]
