# Supplementary material for: Chromosome Specific Substitution Lines of Aegilops geniculata Alter Parameters of Bread Making Quality of Wheat
Source: PLoS One. 2016 Oct 18;11(10):e0162350. doi: 10.1371/journal.pone.0162350 (PMC5068752; doi:10.1371/journal.pone.0162350)
Supplement: S1 Table — (DOCX) [file pone.0162350.s003.docx]

**S1 Table. Comparison of ORF HMW-GS sequences of *Ae. geniculata* with wheat and related sequences.**

| **Subunit** | **Number of amino acid residues** | | | | **Number of cysteine residues** | | | |
| --- | --- | --- | --- | --- | --- | --- | --- | --- |
|  | **N-terminal domain** | **C-terminal domain** | **Repetitive domain** | **Total** | **N-terminal domain** | **C-terminal domain** | **Repetitive domain** | **Total** |
| **1Ux1** | 86 | 42 | 843 | 971 | 3 | 1 | 0 | 4 |
| **1Dx2** | 88 | 42 | 687 | 817 | 3 | 1 | 0 | 4 |
| **1Dx5** | 89 | 42 | 687 | 818 | 3 | 1 | 1 | 5 |
| **1Ax2*** | 86 | 42 | 666 | 794 | 3 | 1 | 0 | 4 |
| **1Bx7** | 81 | 42 | 645 | 768 | 3 | 1 | 0 | 4 |
| **1Ugx** | 86 | 42 | 846 | 974 | 3 | 1 | 0 | 4 |
| **1Mgx** | 84 | 42 | 673 | 799 | 3 | 1 | 0 | 4 |
| **1Mx** | 79 | 42 | 671 | 792 | 3 | 1 | 0 | 4 |
| **1Mgy** | 104 | 42 | 541 | 597 | 5 | 1 | 1 | 7 |
| **1Ugy** | 104 | 42 | 487 | 633 | 5 | 1 | 1 | 7 |
| **1Uy** | 104 | 42 | 425 | 571 | 5 | 1 | 1 | 7 |
| **1My** | 104 | 42 | 451 | 597 | 5 | 1 | 1 | 7 |
| **1Dy10** | 104 | 42 | 481 | 627 | 5 | 1 | 1 | 7 |
| **1Ay** | 104 | 42 | 435 | 581 | 5 | 1 | 0 | 6 |
| **1By9** | 104 | 42 | 538 | 684 | 5 | 1 | 1 | 7 |
