# Supplementary material for: Chromosome Specific Substitution Lines of Aegilops geniculata Alter Parameters of Bread Making Quality of Wheat
Source: PLoS One. 2016 Oct 18;11(10):e0162350. doi: 10.1371/journal.pone.0162350 (PMC5068752; doi:10.1371/journal.pone.0162350)
Supplement: S2 Table — (DOCX) [file pone.0162350.s004.docx]

**S2 Table. Rheological parameters of Green house raised DSLs in comparison to CS (2009-2010) at ICARDA, Syria.**

| **Line** | **TKW** | **Protein** | **PSI** | **FAB** | **FDT** | **FST** | **Softening** |
| --- | --- | --- | --- | --- | --- | --- | --- |
|  | **g** | **%** | **%** | **%** | **min** | **min** | **FU** |
| **1Mg (1A)** | 26.0^b^ | 15.1^a^ | 61.5^a^ | 57.0^a^ | 3.5^d^ | 7.0^c^ | 38.0^d^ |
| **1Mg (1B)** | 26.4^b^ | 14.7^a^ | 57.6^b^ | 57.0^a^ | 3.0^c^ | 3.0^a^ | 85.0^c^ |
| **1Mg (1D)** | 28.5^b^ | 14.2^a^ | 51.0^c^ | 56.0^a^ | 0.9^b^ | 0.9^b^ | 186.0^b^ |
| **CS** | 30.5^a^ | 14.9^a^ | 61.7^a^ | 58.0^a^ | 1.7^a^ | 3.2^a^ | 99.0^a^ |

**Values followed by same letter in same column are not significantly different at p<0.05.**

**FAB- Farinograph absorbance, FDT- Farinograph development time, FST- Farinograph stability time, FU- Farinograph units**
