# Supplementary material for: Chromosome Specific Substitution Lines of Aegilops geniculata Alter Parameters of Bread Making Quality of Wheat
Source: PLoS One. 2016 Oct 18;11(10):e0162350. doi: 10.1371/journal.pone.0162350 (PMC5068752; doi:10.1371/journal.pone.0162350)
Supplement: S3 Table — (DOCX) [file pone.0162350.s005.docx]

| **S3 Table. List of 2D-PAGE spots indicating present (P), absent (A), reduced expression (R) spots in CS, DAL and DSLs** | | | | | | | | |
| --- | --- | --- | --- | --- | --- | --- | --- | --- |
|  |  |  |  |  |  |  |  |  |
| **S. No.** | **Protein** | **CS** | **DAL1Mg** | | **DSL1Mg(1A)** | **DSL1Mg(1B)** | **DSL1Mg(1D)** | |
| 1 | **HMW Glutein** | P | P | | P | P | A | |
| 2 |  | P | P | | P | A | P | |
| 3 |  | P | P | | P | A | P | |
| 4 |  | P | P | | P | P | A | |
| 5 | **ω-gliadin** | P | P | | P | A | P | |
| 6 |  | P | P | | P | A | P | |
| 7 |  | P | P | | P | A | P | |
| 8 |  | P | P | | P | P | A | |
| 9 |  | P | P | | P | P | A | |
| 10 |  | P | P | | P | P | A | |
| 11 | **LMW-GS, α-, γ-gliadin** | P | P | | P | P | P | |
| 12 |  | P | P | | P | A | P | |
| 13 |  | P | P | | A | P | P | |
| 14 |  | P | P | | P | A | P | |
| 15 |  | P | P | | P | R | R | |
| 16 |  | P | P | | P | P | P | |
| 17 |  | P | P | | A | P | R | |
| 18 |  | P | P | | A | A | P | |
| 19 |  | P | P | | P | R | P | |
| 20 |  | P | P | | P | P | A | |
| 21 |  | P | P | | P | P | P | |
| 22 |  | P | P | | P | P | P | |
| 23 |  | P | P | | P | A | A | |
| 24 |  | P | P | | P | P | P | |
| 25 |  | P | P | | P | P | P | |
| 26 |  | P | P | | P | P | P | |
| 27 |  | P | P | | P | P | P | |
| 28 |  | P | P | | P | P | P | |
| 29 |  | P | P | | P | P | A | |
| 30 |  | P | P | | P | P | P | |
| 31 |  | P | P | | P | P | P | |
| 32 |  | P | P | | P | P | P | |
| 33 |  | P | P | | P | P | P | |
| 34 |  | P | P | | P | P | P | |
| 35 |  | P | P | | P | P | P | |
| 36 |  | P | P | | P | A | A | |
| 37 |  | P | R | | R | R | R | |
| 38 |  | P | P | | P | P | A | |
| 39 |  | P | P | | P | A | R | |
| 40 |  | P | P | | P | P | P | |
| 41 |  | P | A | | P | A | A | |
| 42 |  | P | P | | A | A | P | |
| 43 |  | P | P | | P | P | A | |
| ***Ae. geniculata* bands** | | | | | | | | |
| 1 | **HMW Glutenin** | A | | P | P | P | | P |
| 2 |  | A | | P | P | P | | P |
| 3 | **ω-gliadin** | A | | P | P | P | | P |
| 4 |  | A | | P | P | P | | P |
| 5 |  | A | | P | P | P | | P |
| 6 |  | A | | P | P | P | | P |
| 7 |  | A | | P | P | P | | P |
| 8 | **LMW-GS, α-, γ-gliadin** | A | | P | P | P | | P |
| 9 |  | A | | P | P | P | | P |
| 11 |  | A | | x | x | P | | P |
| 12 |  | A | | x | x | P | | P |
| 13 |  | A | | x | x | P | | P |
|  |  |  | |  |  |  | |  |
|  | x indicates bands not detected due to technical problems | | | | | | |  |
